# Clinicopathological and molecular characterization of HPV‐associated cervical poorly cohesive carcinoma: a rare aggressive entity

**DOI:** 10.1002/2056-4538.70100

**Published:** 2026-06-14

**Authors:** Wei Liu, Xiao‐jiang Wang, Yan‐mei Cui, Jing‐cheng Liu, Jiajie Zhang, Li‐bin Zhang, Xi Lei, Xiuqin Weng, Wucheng Shen, Wen‐xiu Miao, Jun‐cheng Ye, Tong‐mei He, Qin Xu, Yi Shi, Dan Hu

**Affiliations:** ^1^ Department of Pathology Clinical Oncology School of Fujian Medical University, Fujian Cancer Hospital Fuzhou PR China; ^2^ Department of Molecular Pathology Clinical Oncology School of Fujian Medical University, Fujian Cancer Hospital Fuzhou PR China; ^3^ Department of Pathology The Affiliated People's Hospital of Fujian University of Traditional Chinese Medicine Fuzhou PR China; ^4^ The School of Basic Medical Sciences Fujian Medical University Fuzhou PR China; ^5^ Medical Records Room Clinical Oncology School of Fujian Medical University, Fujian Cancer Hospital Fuzhou PR China; ^6^ Department of Pathology Xiapu County General Hospital (Xiapu County Hospital) Ningde PR China; ^7^ Department of Pathology Ningde Hospital of Traditional Chinese Medicine, Affiliated to Fujian University of Traditional Chinese Medicine Ningde PR China; ^8^ Department of Gynecology Clinical Oncology School of Fujian Medical University, Fujian Cancer Hospital Fuzhou PR China

**Keywords:** cervical carcinoma, poorly differentiated adenocarcinoma, cervical signet‐ring cell carcinoma, cervical poorly cohesive carcinomas, human papillomavirus

## Abstract

Primary signet‐ring cell carcinoma and poorly differentiated adenocarcinoma with poorly cohesive morphology in the cervix are rare conditions, and their clinicopathological features remain poorly described. This study defines primary cervical poorly differentiated adenocarcinomas meeting the diagnostic criteria for poorly cohesive carcinoma as outlined in the 2019 WHO Classification of Digestive System Tumors as ‘HPV‐associated cervical poorly cohesive carcinomas’ (HPV‐associated CPCC) and describe their clinicopathological and molecular features. Sixteen HPV‐associated CPCC cases were analyzed and classified into three histological subtypes: signet‐ring cell carcinoma (*n* = 4), not otherwise specified (*n* = 6), and mixed types (*n* = 6). All patients were Chinese (median age: 46 years; range: 30–66). Vaginal bleeding was the primary presenting symptom (100.0%). High‐risk human papillomavirus (HPV) was identified in all tumors, with HPV‐18 as the predominant genotype (*n* = 13), HPV‐16 in two cases, and a single case exhibiting concurrent infection with HPV‐16, ‐18, and ‐58. Overall, 56.3% presented with advanced‐stage disease (International Federation of Gynecology and Obstetrics [FIGO] IIIB–IVB), frequently involving regional lymph nodes (56.3%) and distant sites (18.8%). Histopathological examination revealed diffuse stromal infiltration (100%), lymphovascular invasion (75.0%), necrosis (75.0%), and desmoplasia. Immunohistochemically, all cases showed p16 block positivity. Variable expression of antibody‐drug conjugate targets was observed, with HER2‐low expression (33.3%), and positive staining for Trop‐2 (85.7%), nectin‐4 (42.9%), and tissue factor (92.3%). During follow‐up, disease‐specific mortality was 50.0%. The 3‐year overall survival rate was 56.3%, which was significantly lower in advanced‐stage disease (45.0%) than in early‐stage disease (75.0%). Whole‐exome sequencing revealed low tumor mutational burden (median 1.28 Muts/Mb), recurrent mutations in AK1, ARHGAP39, KRT24, MICAL3, SLC6A9 (27.3%), KRAS, and KMT2C (18.2%), alongside MUC2 copy gain (63.6%) and bidirectional Y_RNA alterations (gain 54.5%/loss 45.5%). Collectively, HPV‐associated CPCC represents a distinct and aggressive subtype characterized by distinctive histopathological features, a predominant association with HPV18, frequent presentation at advanced stages, and marked molecular and biomarker heterogeneity.

## Introduction

Cervical cancer remains a significant global health concern, particularly among women in developing countries, where advanced‐stage diagnosis contributes to poor prognosis [1]. Although squamous cell carcinomas account for most cervical cancers, adenocarcinomas constitute a smaller yet steadily increasing proportion of cases [2]. In recent years, a growing body of literature has focused on rare and morphologically distinct subtypes of cervical adenocarcinoma, particularly those exhibiting poorly cohesive growth patterns such as primary cervical signet‐ring cell carcinoma (SRCC) [3]. These tumors often share morphological similarities with poorly cohesive carcinomas of the gastrointestinal tract (particularly of gastric origin), characterized by discohesive growth patterns, frequent lymphovascular invasion, and aggressive clinical behavior. Historically, such tumors have been underreported or misclassified because of their rarity and overlapping morphology with other poorly differentiated malignancies.

The concept of ‘poorly cohesive carcinoma’ is well established in gastrointestinal pathology [4], where it refers to carcinomas composed predominantly of discohesive infiltrating cells, including both signet‐ring cell and non‐signet‐ring cell forms. In the present study, we use the term HPV‐associated cervical poorly cohesive carcinoma (CPCC) as a descriptive study term for HPV‐associated endocervical adenocarcinomas exhibiting a predominant poorly cohesive infiltrative pattern, with the goal of systematically characterizing their clinicopathological and molecular features and providing a basis for a more comprehensive understanding of this lesion among pathologists and gynecologic oncologists.

This study aimed to address this knowledge gap by systematically analyzing 16 patients with HPV‐associated CPCC, supplemented by a literature review. The objectives were to describe the clinicopathological features of HPV‐associated CPCC, including histologic subtypes, HPV status, and survival outcomes; assess its immunohistochemical profile, including E‐cadherin, p53, mismatch repair (MMR) proteins, and PD‐L1; evaluate potential therapeutic targets such as HER2, trophoblast cell‐surface antigen 2 (Trop‐2), nectin‐4, and tissue factor (TF); and characterize its genomic and proteomic features. Through this approach, we sought to provide a framework for understanding HPV‐associated CPCC as a distinct variant of cervical cancer with potential implications for classification, clinical management, and targeted therapy.

## Materials and methods

The study protocol was conducted in accordance with the Declaration of Helsinki and approved by the Institutional Ethics Committee of Fujian Cancer Hospital (approval no. K2024‐051‐01). All patients provided written informed consent for the use and publication of their medical data at their first hospital visit.

### Diagnostic criteria for HPV‐associated CPCC

The diagnostic criteria for HPV‐associated CPCC were based on the 2019 WHO Classification of Digestive System Tumors and the 2019 Consensus Statement on the Pathological Definition and Classification of Poorly Cohesive Gastric Carcinoma [4, 5]. A signet‐ring cell was defined as a cell with ample cytoplasmic mucin that appears optically clear on hematoxylin and eosin (H&E) staining, with an eccentrically placed nucleus [5]. All other poorly cohesive cancer cells lacking this specific morphology were classified as poorly cohesive cells, not otherwise specified (NOS). WHO poorly cohesive carcinomas with more than 90% possessing signet‐ring cell morphology were classified as the SRCC subtype. All other poorly cohesive non‐SRCC subtypes were further subdivided into HPV‐associated CPCC with SRC components (<90% but >10% signet‐ring cells) and HPV‐associated CPCC NOS subtypes (<10% signet‐ring cells). Tumors were classified as mixed if the poorly cohesive carcinoma component accounted for ≥10% but <90% of the entire tumor. Cases in which the poorly cohesive carcinoma portions constituted <10% of the entire tumor were excluded. Other rare cervical carcinomas, including poorly differentiated neuroendocrine carcinoma, poorly differentiated squamous cell carcinoma, carcinosarcoma, and undifferentiated carcinoma, were also excluded.

### Case selection

This study included a retrospective consecutive cohort of 2,500 patients with cervical adenocarcinoma who underwent initial surgical resection or biopsy at Fujian Cancer Hospital between 2009 and 2024. H&E‐stained slides containing tumor tissues from each case were examined using a multiheaded microscope, and consensus diagnoses were reached, following a slide review by three pathologists (WL, DH, and Y‐mC). Twenty‐two cases met the histological diagnostic criteria for poorly cohesive carcinoma. All patients underwent HPV PCR testing and immunohistochemistry (IHC) staining for p16. p16 positivity was defined as diffuse and strong nuclear and cytoplasmic staining (block‐positive p16 reactivity), while p16 negativity was defined as focal/patchy staining or complete absence of staining. Additionally, all patients underwent comprehensive gastrointestinal endoscopy and imaging evaluation of the hepatobiliary–pancreatic system, including MRI, CT, PET‐CT, and ultrasonography. Among them, six cases that were negative for both HPV and p16 were excluded because occult metastatic carcinomas of the gastrointestinal tract could not be ruled out, despite the absence of detectable lesions in other organs on imaging studies. The remaining 16 patients showed no abnormalities in the gastrointestinal tract, hepatobiliary system, pancreas, breast, or other relevant organs, and exhibited synchronous positivity for both high‐risk HPV and p16. Ultimately, these 16 patients were enrolled in the study cohort as HPV‐associated CPCC.

### Morphologic evaluation

A comprehensive assessment of histological features, including tumor margin, size, necrosis, lymphovascular invasion, growth pattern, and cell morphology, was conducted to classify tumors into distinct morphological groups. The International Federation of Gynecology and Obstetrics (FIGO) stage, lymph node metastasis status, involvement of other organs, and distant metastasis status were also documented for all cases.

### Immunohistochemical staining

Formalin‐fixed, paraffin‐embedded tissue blocks were sectioned at 4 μm and stained on a Ventana BenchMark ULTRA autostainer (Roche, Switzerland). Antibodies included epithelial, site‐associated, and therapeutic markers (E‐cadherin, p16, PAX8, CK7, CK19, CK20, Villin, CDX2, SATB2, MUC6, Claudin 18.2, CK5/6, p40, p53, PD‐L1, HER2, TROP2, nectin‐4, and TF), as well as MMR proteins (MLH1, PMS2, MSH2, and MSH6). Positive and negative controls were included in each run. HER2 was scored according to the 2017 CAP/ASCP/ASCO gastric cancer guidelines [6]. PD‐L1 expression was assessed using the combined positive score and classified as negative (<1), low (1–49), or high (≥50). p53 staining was interpreted as mutant‐type (diffuse strong nuclear staining in ≥80% of tumor cells, complete absence of nuclear staining, or cytoplasmic staining) or wild‐type (heterogeneous nuclear staining). TROP2, nectin‐4, and TF expression was evaluated using the H‐score (range, 0–300) and categorized as 0 (<10), 1+ (10–99), 2+ (100–199), or 3+ (200–300) [7].

### HPV‐PCR testing

HPV detection and genotyping were conducted on FFPE tissue sections using the AmoyDx HPV Nucleic Acid Detection Kit (Lot: 8.01.25803W048A) according to the manufacturer's protocol. This real‐time PCR‐based assay targets the HPV L1 gene with genotype‐specific primers/probes, detecting 17 high‐risk types (16, 18, 26, 31, 33, 35, 39, 45, 51, 52, 53, 56, 58, 59, 66, 68, 82) and 2 low‐risk types (6, 11).

### Whole‐exome sequencing (WES) analysis

WES was performed on 11 paired samples of HPV‐associated CPCC and adjacent nontumor tissues using the Agilent SureSelect Human All Exon V8 capture platform. High‐throughput sequencing was conducted using the MGI MGISEQ‐T7 instrument. Variant calling followed the GATK best practices pipeline. Somatic mutations were identified via Mutect2, which employs local realignment of mutation hotspots and Bayesian statistical modeling for paired tumor‐normal analysis, followed by stringent filtration to remove variants present in matched normal samples or the dbSNP database. Functional annotation of SNPs/indels was executed using VEP, while copy number variations (CNVs) were quantified with CNVkit under default settings.

### Proteomics workflow

Proteins were extracted in 8 M urea/50 mM Tris–HCl buffer with protease inhibitors, followed by homogenization, centrifugation, reduction, and alkylation. Protein concentration and quality were assessed by Bradford assay and SDS‐PAGE, respectively. For each sample, 150 μg of protein was digested with trypsin, desalted, and dried. Peptides from each sample were fractionated separately by high‐pH reversed‐phase chromatography into 10 fractions. Each fraction was reconstituted in 0.1% formic acid and analyzed by LC–MS/MS on a Thermo Astral instrument in DIA mode. Raw data were processed using DIA‐NN (v1.9.1) with a library‐free search against the UniProt database. Protein intensities were median‐normalized and imputed with minimal values in R (version 4.0). Differentially expressed proteins (DEPs) were identified using Student's *t*‐test with fold change >1.2 and *p* < 0.05. Gene Ontology (GO) enrichment analysis was performed using a hypergeometric test, with *p* < 0.05 considered statistically significant.

### Statistical and survival analysis

The follow‐up duration was calculated from the date of diagnosis based on the cervical sample to death or last contact. Analyses were performed using SPSS software (version 23.0; SPSS Inc., New York, NY, USA). Survival duration was assessed using Kaplan–Meier analysis. Statistical significance was set at *p* < 0.05.

## Results

### Patients' clinical characteristics

All 16 patients were Chinese, with a median age of 46 years (range: 30–66 years). Vaginal bleeding was the predominant presenting symptom in all cases, while one case was accompanied by dull lower abdominal pain. Among the cohort, four patients were postmenopausal. High‐risk HPV infection was detected in all 16 cases (100.0%), predominantly HPV type 18 (13 cases, 81.3%) and type 16 in 2 cases. One patient exhibited co‐infection with HPV types 16, 18, and 58. Tumor marker analysis revealed elevated CEA levels in 11/16 cases (median: 45.0 ng/ml), elevated CA125 in 7/16 cases (median: 144.0 U/ml), and elevated CA199 in 7/16 cases (median: 110.0 U/ml). According to the 2018 FIGO staging system, cases ranged from stage IB2 to IVB, with stage IIIB–IVB comprising 56.3% (9/16) of cases. TNM staging demonstrated regional lymph node metastasis in nine cases (56.3%) and distant metastasis (M1) in three cases (18.8%). The clinical data are presented in Table 1.

**Table 1:** Clinicopathological characteristics of HPV‐associated CPCC

Case	Age	Presentation	Post‐menopause	HPV (PCR)	CEA (ng/ml)	CA125 (U/ml)	CA199 (U/ml)	Tumor size (cm)	Primary tumor (T)	Lymph node (N)	Distant metastasis (M)	Other organ involvement	Stage	Treatment	Follow‐up*
1	60	VB	Yes	HPV 18	63.4	217	115	5	T2b	N0	M0	None	IIB	Biopsy, CT + RT	7 months, DOD
2	52	Postcoital VB	No	HPV 18	ND	53	110	4.7	T3b	N1	M0	None	IIIC1	Biopsy, CT + RT	7 months, DOD
3	48	VB	No	HPV 18	30.5	ND	ND	3	T1b2	N0	M0	None	IB2	ST + CT	80 months, ANED
4	52	Abdominal pain with VB	No	HPV 18	ND	900.5	249.4	7	T3b	N2	M1 (left cervical LN)	Right ovary	IVB	ST + CT + IT + TT	26 months, DOD
5	36	VB	No	HPV 18	ND	ND	ND	6	T3b	N1	M0	None	IIIC1	Biopsy, CT + RT	20 months, DOD
6	46	VB	No	HPV 18	34	14.5	86	8	T2b	N1	M0	None	IIIC1	Biopsy, CT + RT	6 months, DOD
7	30	Postcoital VB	No	HPV 16	25	ND	ND	3	T2a1	N0	M0	None	IIA1	ST + CT	44 months, AWD
8	66	VB	Yes	HPV 16, 18, 58	45	ND	ND	3.5	T2a1	N0	M0	None	IIA	Biopsy, CT + RT	36 months, AWD
9	46	VB	No	HPV 18	7,105	144	51.5	4.1	T2a2	N1	M0	None	IIIC1	ST + CT + RT + TT	40 months, DOD
10	63	VB	Yes	HPV 18	98.3	247.1	168.5	5	T3b	N1	M1 (bilateral cervical LN)	None	IVB	Biopsy, CT + RT	12 months, AWD
11	57	VB	Yes	HPV 18	549	63.5	7	3.9	T1b2	N1	M0	None	IIIC1	Biopsy, CT + TT	59 months, DOD
12	47	Postcoital VB	No	HPV 18	2.5	ND	ND	4	T2a1	N1	M0	None	IIIC1	ST + CT	48 months, AWD
13	40	VB	No	HPV 18	118	194.4	2,240	7	T3b	N2a	M1 (left cervical and left inguinal LN)	None	IVB	Biopsy, CT + TT	2 months, AWD
14	42	Postcoital VB	No	HPV 18	40.4	3	5	3	T1b2	N0	M0	None	IB2	ST + CT	3 months, ANED
15	47	VB	No	HPV 18	ND	ND	ND	5	T2b	N0	M0	None	IIB	Biopsy, CT + RT	LTF
16	39	Postcoital VB	No	HPV 16	50.5	ND	ND	2.5	T1b2	N0	M0	None	IB2	ST + CT	LTF

ANED, alive with no evidence of disease; AWD, alive with disease; CT, chemotherapy; DOD, dead of disease; IT, immunotherapy; LTF, lost to follow up; ND, not done; RT, radiotherapy; ST, surgical treatment (total hysterectomy with bilateral adnexectomy, pelvic lymph node dissection, and para‐aortic lymph node dissection); TT, targeted therapy; VB, vaginal bleeding.

* Follow‐up time was calculated from the date of diagnosis of HPV‐associated CPCC.

### Pathological features

CPCC exhibited tumor sizes ranging from 2.5 to 8.0 cm (median: 4.7 cm), with eight (50.5%) cases demonstrating parametrial involvement. Histologically, these tumors comprised isolated neoplastic cells or small aggregates lacking well‐formed glandular structures. Two distinct morphological patterns were identified: signet‐ring cells characterized by central optically clear cytoplasmic mucin droplets displacing nuclei eccentrically, and non‐signet‐ring cells resembling histiocytes or lymphocytes. Based on the diagnostic criteria for HPV‐associated CPCC, four SRCC, six NOS, and six mixed subtype cases were identified. IHC analysis revealed block‐positive p16 reactivity for all 16 HPV‐positive cases. Necrosis of variable extent was observed in 75.0% (12/16) of the tumors, while lymphovascular space invasion was identified in 75.0% (12/16) of the cases, categorized as focal (*n* = 9) or substantial (*n* = 3). These results are summarized in Table 2.

**Table 2:** Histological features and classification of HPV‐associated CPCC

Case	SRC component (%)	NOS components (%)	Other adenocarcinoma components (%)	Tumor stromal mucinous change	Tumor giant cells	Mitosis (per 10 HPF)	Necrosis	Lympho‐vascular invasion	Histologic subtype
1	100%	0%	0%	No	No	3	None	Focal	SRCC subtype
2	100%	0%	0%	Yes	No	3	Focal	No	SRCC subtype
3	100%	0%	0%	Yes	No	5	Focal	Substantial	SRCC subtype
4	95%	0%	5%, UCA	Yes	Yes	10	Focal	Substantial	SRCC subtype
5	0%	100%	0%	No	No	5	Diffuse	Focal	NOS subtype
6	0%	100%	0%	No	No	10	Focal	Focal	NOS subtype
7	0%	100%	0%	No	No	10	Focal	No	NOS subtype
8	0%	100%	0%	No	No	5	Diffuse	No	NOS subtype
9	0%	100%	0%	No	No	10	Focal	Substantial	NOS subtype
10	5%	95%	0%	No	No	10	Focal	No	NOS subtype
11	20%	50%	30%, UCA	No	No	5	Focal	Focal	Mixed (CPCC with SRC component and UCA)
12	30%	40%	30%, ISMC	Yes	No	10	None	Focal	Mixed (CPCC with SRC component and ISMC)
13	70%	0%	30%, ISMC	Yes	No	10	Diffuse	Focal	Mixed (SRCC subtype and ISMC)
14	50%	0%	50%, ISMC	Yes	Yes	10	Focal	Substantial	Mixed (SRCC subtype and ISMC)
15	80%	0%	20%, ISMC	Yes	No	5	None	Focal	Mixed (SRCC subtype and ISMC)
16	0%	60%	40%, UCA	No	No	5	None	Focal	Mixed (NOS subtype and UCA)

CPCC, cervical poorly cohesive carcinomas; ISMC, invasive stratified mucin‐producing carcinoma; NOS, not otherwise specified; SRC, signet‐ring cell; SRCC, signet‐ring cell carcinoma; UCA, usual‐type cervical adenocarcinoma.

#### HPV‐associated CPCC, SRCC subtype (*n* = 4)

**Figure 1:**
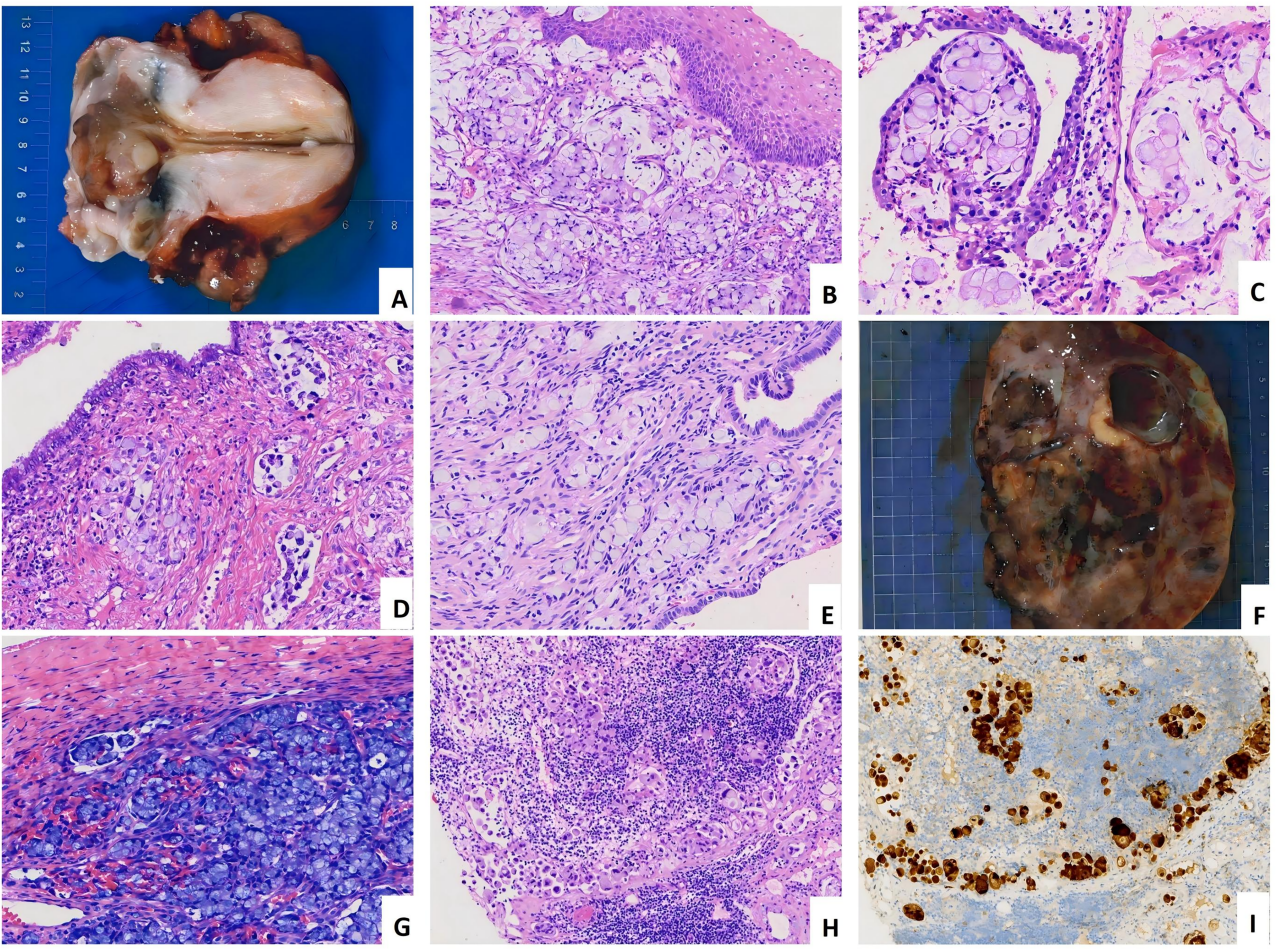
HPV‐associated cervical poorly cohesive carcinomas (CPCC), signet‐ring cell carcinoma subtype. (A) Invasive cervical mass with grayish‐white cut surfaces exhibiting a soft, delicate texture. (B–D) Tumor cells display signet‐ring cell morphology, infiltrating cervical stroma with evident lymphovascular invasion. (E) Cancer cells invading the endometrial stroma. (F and G) Ovarian metastasis: The cut surface appears gray‐white with fish‐flesh texture, showing marked hemorrhage and cystic degeneration; tumor cells infiltrate the ovarian stroma. (H and I) Metastasis to cervical lymph nodes, confirmed by P16‐positive.

Grossly, these tumors presented as nodular masses exhibiting invasive growth, infiltrating the full cervical stroma thickness, with cut surfaces appearing grayish‐white and possessing a soft, delicate texture (Figure 1A). Microscopically, tumor cells diffusely infiltrated the cervical stroma, accompanied by a mild‐to‐moderate desmoplastic stromal reaction; stromal mucinous changes were observed in three cases. Neoplastic cells displayed characteristic signet‐ring cell morphology, featuring abundant cytoplasm and eccentrically placed hyperchromatic nuclei (Figure 1B,C). Mitotic activity ranged from 2 to 10 figures per 10 high‐power fields (HPFs), with a median of 5/10 HPF. Focal necrosis was evident in three cases, and lymphovascular invasion in three cases (Figure 1D), two of which were substantial. Endometrial tumor involvement was documented in two cases (Figure 1E). Case 4 had right ovary metastasis (maximum diameter, 12 cm) with cut surface being solid, grayish‐white, and nodular. Carcinoma cells were mucin‐rich, infiltrating the stroma (Figure 1F,G). Additionally, case 4 also exhibited left cervical (neck) lymph node metastasis, where tumor cells exhibiting signet‐ring cell morphology consistent with the primary cervical lesion were identified within the nodal sinuses. IHC analysis demonstrated p16 positivity, which was corroborated by HPV‐PCR testing, confirming HPV type 18 infection (Figure 1H,I).

#### HPV‐associated CPCC, NOS subtype (*n* = 6)

**Figure 2:**
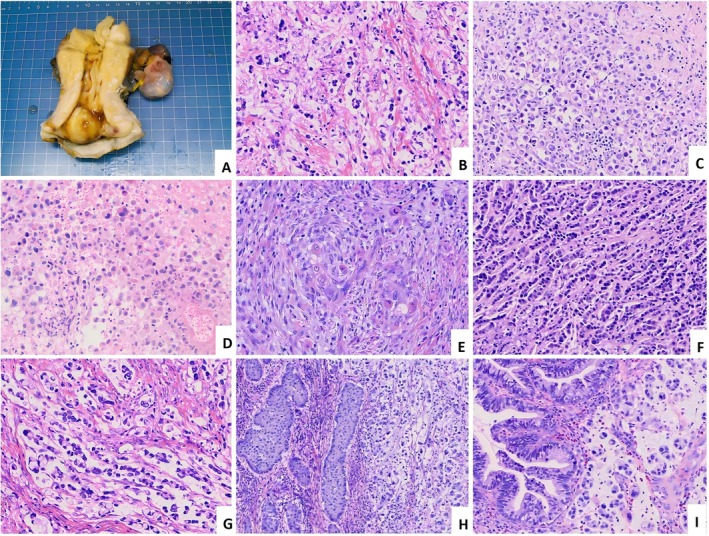
HPV‐associated cervical poorly cohesive carcinomas (CPCC), NOS subtype (A–G), and mixed subtype (H and I). (A) A raised mass is visible in the cervix. (B) The neoplastic cells demonstrate low cohesiveness, predominantly arranged as single cells. (C) Lymphoma‐like tumor cells, with visible tumor thrombus. (D) Histiocyte‐like tumor cells. (E) Scattered single eosinophilic tumor cells in desmoplastic stroma. (F) Tumor cells in an ‘Indian filing’ pattern (referring to a single‐file linear arrangement). (G) Tumor cells mixed with features of signet‐ring cell carcinoma. (H) Invasive stratified mucin‐producing carcinoma (ISMC) component (left); (I) Conventional cervical adenocarcinoma component (left).

Tumors exhibited a nodular configuration, infiltrating the cervical stroma with deep‐to full‐thickness involvement. Upon sectioning, the lesion displayed a gray‐white cut surface and firm consistency (Figure 2A). Histopathological examination revealed diffuse infiltration of the cervical stroma. The neoplastic cells demonstrated low cohesiveness, appearing lymphocyte‐like (Figure 2B,C), or histiocyte‐like (Figure 2D,E), predominantly arranged as single cells, small clusters, or cords/linear chains (‘Indian filing’) (Figure 2F), with no evidence of solid sheets or glandular formation. A prominent desmoplastic stromal reaction was consistently present, accompanied by the infiltration of lymphocytes and plasma cells of varying densities, with occasional stromal mucinous changes. The tumor cells were of medium size, displaying round, oval, or polygonal morphology. The cytoplasm was eosinophilic, while nuclei were hyperchromatic with significant atypia, and 4/6 cases showed histiocyte‐like tumor cells. Nucleoli were inconspicuous or small, and mitotic figures were readily identifiable (consistently ≥5 HPF); SRCC foci were interspersed within the tumor in one case (Figure 2G). Four cases had lymph node metastasis, with one case involving bilateral cervical lymph nodes.

#### HPV‐associated CPCC, mixed subtype (*n* = 6)

Six cases of mixed‐type CPCC were identified: four cases contained an invasive stratified mucin‐producing carcinoma component comprising 20–50% of tumor volume (Figure 2H); the remaining two cases contained a usual‐type cervical adenocarcinoma (UCA) component, accounting for 30% and 40% of tumor volume, respectively (Figure 2I).

### Immunophenotypic characteristics

**Figure 3:**
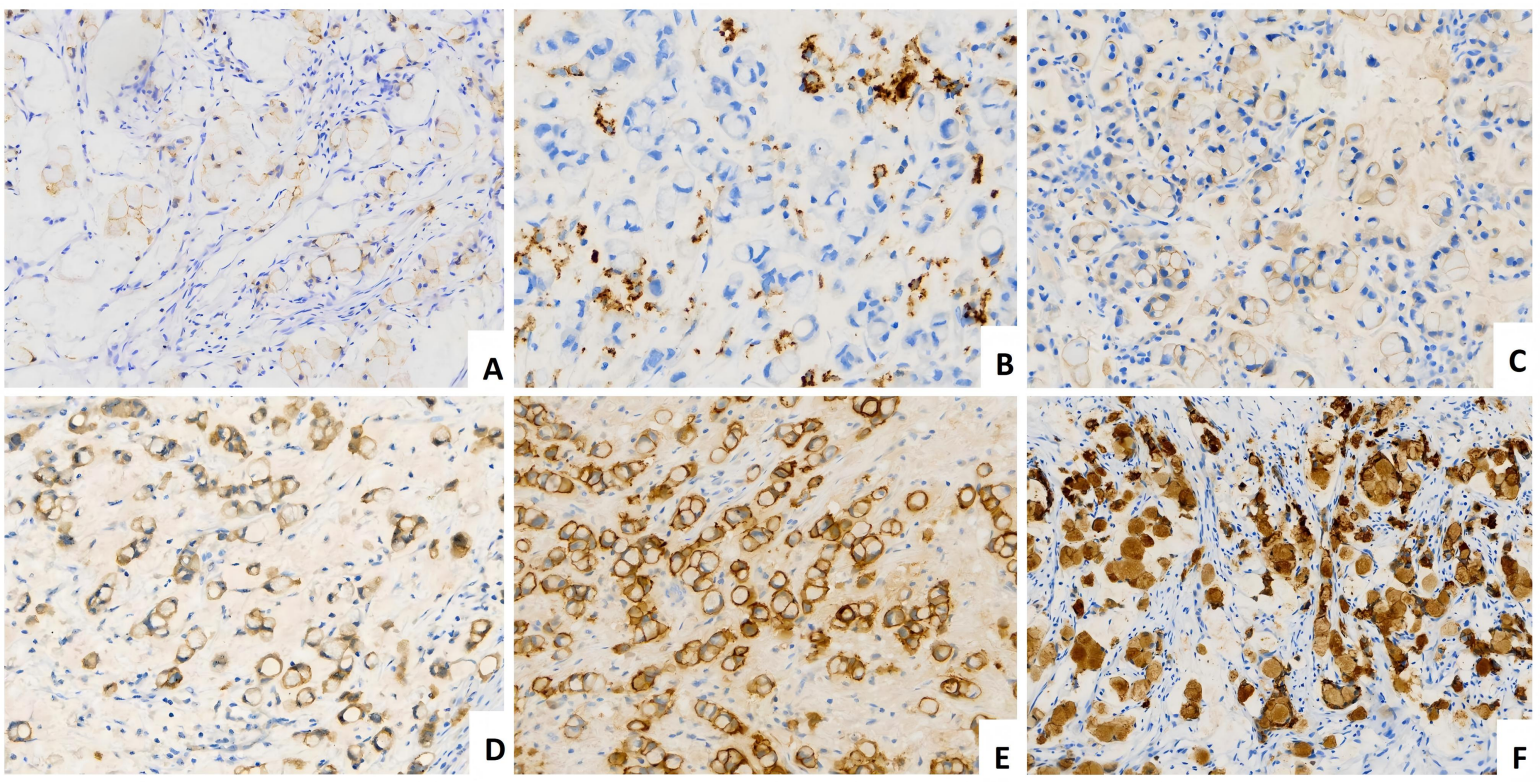
Immunohistochemical features of HPV‐associated cervical poorly cohesive carcinomas (CPCC). (A) Tumor cells show faint expression of E‐cadherin. (B) PD‐L1 shows partial positivity in immune cells. (C) HER2 2+. (D) Nectin‐4 2+. (E) TROP2 3+. (F) TF 3+.

IHC analysis of 16 cases revealed diminished E‐cadherin staining in 8 cases (50.0%) (Figure 3A), and p16 block positivity in all cases. Pax‐8 staining showed diffuse positivity in only one case and focal positivity in two cases, with the remaining 13 cases (81.3%) being negative. Positive staining rates were as follows: CK7, 90.0% (10/11); CK20, 11.1% (1/9); Villin, 26.7% (4/15); CDX‐2, 33.3% (3/9); SATB‐2, 21.4% (3/14); and MUC6, 28.6% (4/14); Claudin 18.2, CK5/6, and p40 were uniformly negative across all assessed cases. p53 IHC staining demonstrated mutant‐type expression in 2 of 16 cases (12.5%), while the remaining 14 (87.5%) exhibited wild‐type expression patterns. MMR protein assessment revealed loss of MSH6 expression in 1 of 14 evaluable cases (MMR deficiency, 7.1%), while all other MMR proteins (MLH1, PMS2, and MSH2) were retained; PD‐L1 (CPS ≥1) was positive in 5 cases (31.3%), with scores ranging from 1 to 30 (median = 5) (Figure 3B). Based on antibody‐drug conjugate (ADC)‐related detection, the target expression profiles across four biomarkers were as follows: HER2 was 2+ in 20.0% (3/15) with all FISH tests negative, 1+ in 13.3% (2/15), and negative in 66.7% (10/15) (Figure 3C); Trop‐2 was 3+ in 64.3% (9/14), 2+ in 21.4% (3/14) (Figure 3D), and negative in 14.3% (2/14); nectin‐4 was 2+ in 42.9% (6/14), and negative in 57.1% (8/14) (Figure 3E); while TF was 3+ in 76.9% (10/13), 2+ in 15.4% (2/13), and negative in 7.7% (1/13) (Figure 3F), reflecting significant heterogeneity in target distribution. The immunophenotypic characteristics are presented in Table 3.

**Table 3:** Immunohistochemical features of HPV‐associated CPCC

Case	E‐cadherin	P16	PAX8	CK7	CK20	Villin	CDX‐2	SATB2	MUC6	Claudin 18.2	CK5/6	P40	P53	dMMR	PDL‐1 (CPS)	HER2	Nectin‐4	TF	TROP2
1	+, W	+	−	+	−	+	−	−	−	−	ND	ND	WT	Yes	0	0	0	3+	0
2	+, S	+	−	+	−	−	+	−	+	−	−	−	WT	No	0	0	0	3+	2+
3	+, W	+	−	−	+	+	+	+	+	−	ND	ND	MT	No	0	0	0	3+	2+
4	+, W	+	+	+	−	−	+	−	+	−	ND	−	WT	No	20	2+	0	3+	3+
5	+, W	+	−	+	−	−	−	+	−	−	ND	−	WT	No	0	0	0	3+	0
6	+	+	−	+	ND	−	−	+	−	−	−	−	WT	No	0	0	2+	2+	3+
7	+, M	+	+, F	+	ND	−	ND	−	−	−	−	−	WT	No	5	0	0	0	3+
8	+, M	+	−	+	ND	ND	ND	ND	ND	ND	−	−	WT	ND	0	ND	ND	ND	ND
9	+, M	+	−	+	ND	−	ND	−	−	−	−	−	WT	No	0	0	2+	2+	3+
10	+, S	+	−	+	ND	+	ND	ND	−	ND	−	−	WT	ND	0	2+	ND	ND	ND
11	+, S	+	−	+	ND	−	ND	−	−	−	−	−	WT	No	0	0	2+	3+	3+
12	+, W	+	−	+	−	−	−	−	+	−	−	ND	WT	No	1	1+	2+	3+	3+
13	+, S	+	−	+	−	+	−	−	−	−	ND	ND	MT	No	5	1+	2+	3+	2+
14	+, S	+	−	+	−	−	ND	−	ND	−	−	−	WT	No	30	2+	2+	ND	3+
15	+, S	+	−	+	−	−	−	−	−	−	ND	ND	WT	No	0	0	0	3+	3+
16	+, S	+	+, F	ND	ND	−	ND	−	−	−	ND	ND	WT	No	0	0	0	3+	3+

+, positive; −, negative; CPCC, cervical poorly cohesive carcinomas; F, focal; M, moderate; MT, mutant‐type expression; ND, not done; S, strong; W, weak; WT, wild‐type expression.

### Treatment and outcomes

**Figure 4:**
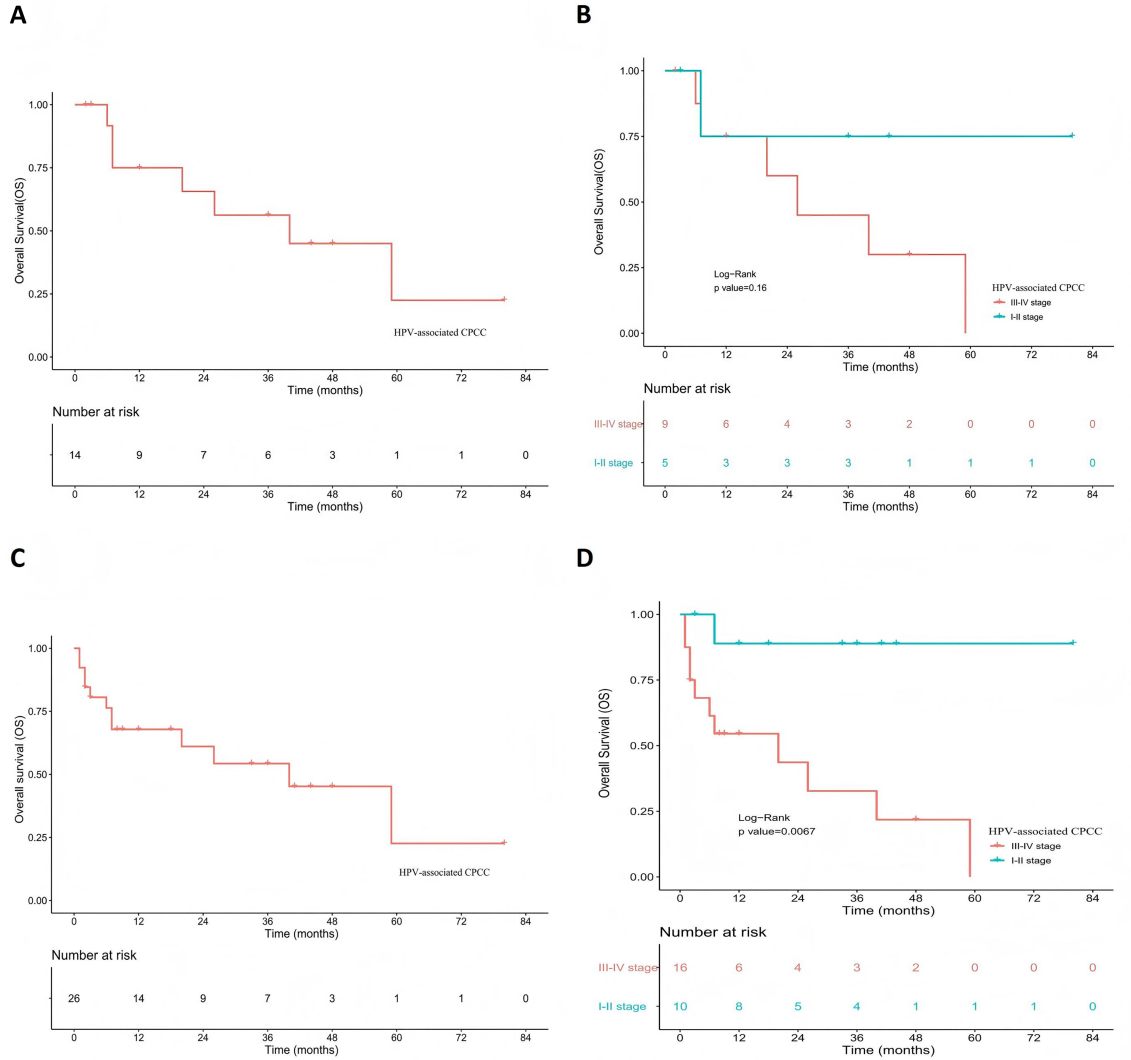
The survival analysis of patients with HPV‐associated cervical poorly cohesive carcinomas (CPCC). (A) Overall survival (OS) analysis of patients with CPCC in the present study (*n* = 14). (B) OS analysis of patients with stage III–IV and stage I–II of CPCC in the present study (*n* = 14). (C) OS analysis of patients with CPCC in the present study and literature (*n* = 28). (D) OS analysis of patients with stage III–IV and stage I–II of CPCC in the present study and literature (*n* = 28).

Seven patients received chemotherapy combined with radiotherapy, and two received chemotherapy combined with targeted therapy. Another seven patients underwent radical surgery (total hysterectomy with bilateral adnexectomy, pelvic and para‐aortic lymph node dissection, with or without omentectomy) followed by postoperative adjuvant therapy (including chemotherapy, radiotherapy, immunotherapy, or targeted therapy). Follow‐up data for the survival analysis were available for 14 patients. Over a median follow‐up period of 26 months (range: 2–80 months), outcomes included death due to disease in 50.0% (7/14), alive with disease (AWD) in 35.7%, and alive with no evidence of disease in 14.3% (2/14), indicating a significant correlation between advanced disease stage and poor prognosis. The 3‐year overall survival (OS) rate was 56.3%; for FIGO stage III–IV patients, the 3‐year OS was only 45.0%, compared to 75.0% for stage I–II patients (Figure 4A,B).

### Review of 28 cases of HPV‐associated CPCC

After an extensive survey of English literature published between 1990 and 2025, we identified 12 reported cases of HPV‐associated CPCC from 11 publications [3, 8, 9, 10, 11, 12, 13, 14, 15, 16, 17]. Inclusion criteria were: sufficiently complete clinicopathological data, including clear pathological images. Cases were excluded if pathological images did not meet the morphological diagnostic criteria for HPV‐associated CPCC. Detailed clinical data for these cases are provided in supplementary material, Table S1. Combined evaluation of these cases with our 16 institutional cases showed a median patient age of 47 years (range: 30–68 years), with vaginal bleeding reported in 85.2% (23/27) of patients. High‐risk HPV infection was predominantly HPV‐18 (85.0%, 17/20). Tumors exceeding 4 cm were found in 48.0% (12/25) of cases. Based on the 2018 FIGO staging system, 42.9% (12/28) were classified as stage I–II and 57.1% (16/28) as stage III–IV; distant metastasis occurred in 25.0% (7/28) of patients, including five with cervical lymph node metastasis and two lung metastasis cases; four others showed pelvic organ involvement. A summary of clinicopathological features is presented in Table 4. Follow‐up data for 26 patients, spanning 1–80 months (median: 12 months), revealed a disease‐related mortality rate of 46.2% (12/26), whereas 23.1% (6/26) survived the disease, resulting in a 3‐year OS of 54.3%. Advanced‐stage tumors had markedly worse prognosis, with FIGO III–IV carcinomas exhibiting a drastically lower 3‐year OS of 32.7%, in stark contrast to the 88.9% observed in early stage (I–II) disease (*p* = 0.0067) (Figure 4C,D).

**Table 4:** A summary of the clinicopathological features of HPV‐associated CPCC from the present study and the literature

Characteristics	Present study	Literature	Total
Number of cases	16	12	28
Median age (range) (years)	46 (30–66)	43 (31–68)	47 (30–68)
Presentation vaginal bleeding (yes/no)	16/0	7/4	23/4
HPV (18+/16+)	13/2	4/1	17/3
Tumor size (≤4 cm/>4 cm)	7/9	6/3	13/12
FIGO 2018 stage (I–II/III–IV)	7/9	5/7	12/16
Distant metastasis (yes/no)	3/13	4/8	7/21
Pelvic organs involvement (yes/no)	1/15	3/9	4/24
Median follow‐up (range) (months)	26 (2–80)	9 (1–41)	12 (1–80)
Outcome (died/alive)	7/7	5/7	12/14

+, positive; CPCC, cervical poorly cohesive carcinomas; FIGO, International Federation of Gynecology and Obstetrics.

### Whole‐exome sequencing revealed the molecular characteristics of HPV‐associated CPCC

WES was performed on 22 FFPE specimens, comprising 11 HPV‐associated CPCC samples and their 11 matched adjacent noncancerous tissues. Each sample generated an average of 27.7 Gb of sequencing data, with 96.8% of the target regions achieving a minimum coverage depth of 100×. After quality filtering (Q30 threshold), the mean base quality score was 95.8% (range: 95.1–98.3%), meeting all requirements for downstream bioinformatic analyses. Overall, HPV‐associated CPCC exhibited a low tumor mutation burden for SNV/Indel, with a median of 1.28 Muts/Mb (range: 0.14–8.69 mut/Mb).

#### Mutation landscape and major mutant genes

**Figure 5:**
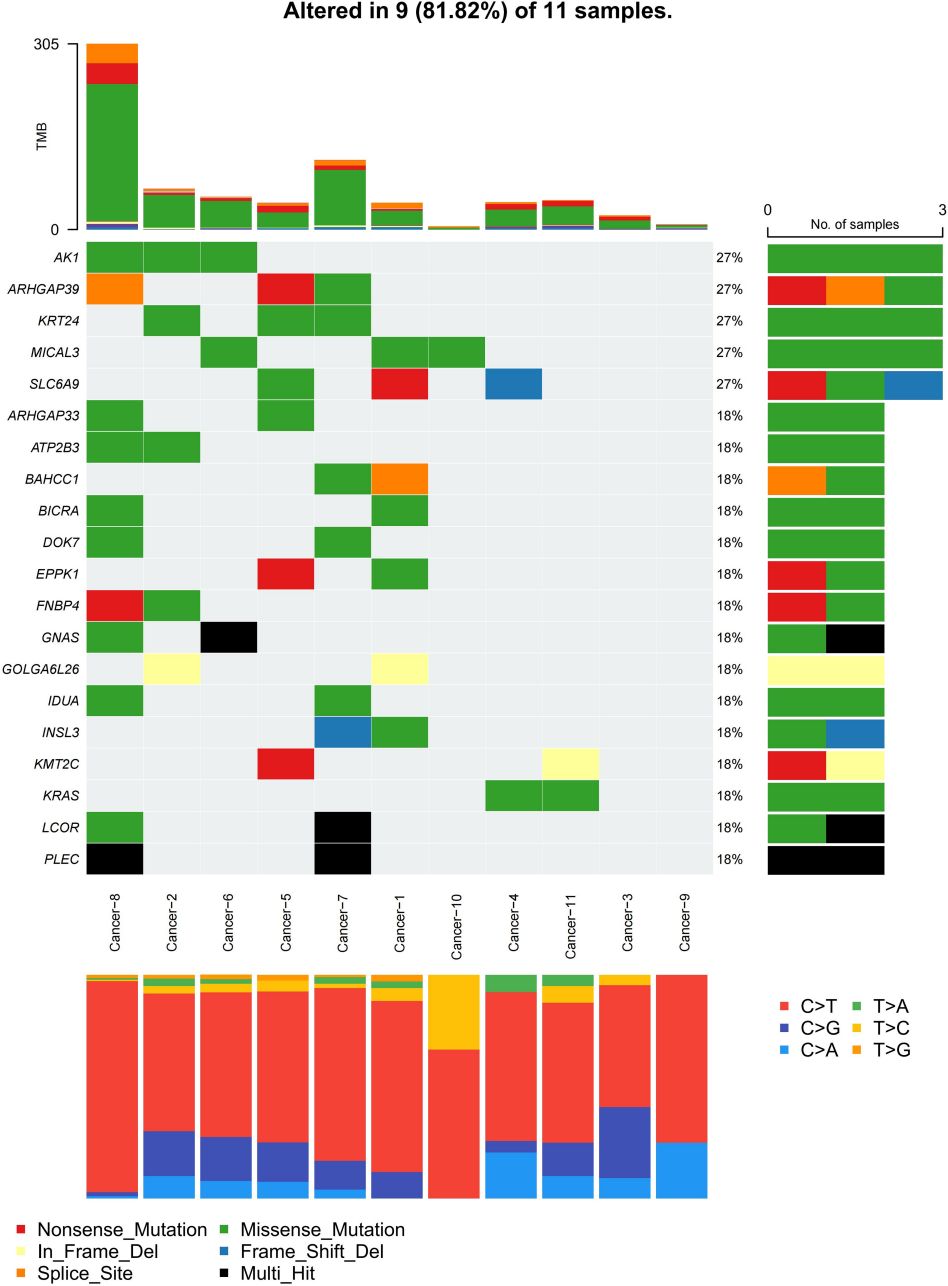
The molecular characteristics of HPV‐associated cervical poorly cohesive carcinomas (CPCC).

Genomic landscape analysis of 11 HPV‐associated CPCC cases, visualized using a waterfall plot, revealed somatic alterations in 81.8% (9/11) of specimens. Among the 20 genes with mutation frequencies ≥18%, the most recurrently mutated genes were AK1, ARHGAP39, KRT24, MICAL3, and SLC6A9 (each in 27.3%, 3/11 cases), followed by ARHGAP33, ATP2B3, BAHCC1, BICRA, DOK7, EPPK1, FNBP4, GNAS, GOLGA6L26, IDUA, INSL3, KMT2C, KRAS, LCOR, and PLEC (each in 18.8%, 2/11 cases). Missense mutations were the predominant alteration type, with additional variants including nonsense mutations, in‐frame deletions, splice site alterations, frameshift deletions, and multi‐hit events. Pathway enrichment analysis implicated these high‐frequency genes in oncogenic processes: AK1 modulates adenosine metabolism, KRAS drives RAS/RAF signal transduction, and KMT2C regulates epigenetic modifications. This molecular heterogeneity highlights the complex biological mechanisms underlying HPV‐associated CPCC pathogenesis. The mutation landscape of HPV‐associated CPCC is shown in Figure 5.

#### Copy number variation

CNV analysis of cancer‐related genes in 11 HPV‐associated CPCC samples revealed distinct genomic alteration patterns: MUC2 exhibited the highest gain frequency (63.6%, 7/11), followed by Y_RNA (54.5%, 6/11), while MUC12, MUC4, Metazoa_SRP, SNORA70, and ANKRD36C each showed gains in 36.4% (4/11) of cases, with CEACAM8, CEACAMP9, and ENSG00000227606 each in 27.3% (3/11). Conversely, loss events were most frequent in Y_RNA (45.5%, 5/11), while ENSG00000260605, ENSG00000260620, ENSG00000260850, ENSG00000261047, ENSG00000280344, ENSG00000285367, ENSG00000287256, ENSG00000287718, and HNRNPA1P48 all demonstrated losses in 36.4% (4/11) of cases, alongside Metazoa_SRP (27.3%, 3/11) and SNORA70 (9.1%, 1/11) (supplementary material, Figure S1).

### Mutational landscape of HPV‐associated usual‐type endocervical adenocarcinoma (UECA) in TCGA and comparative analysis with HPV‐associated CPCC

Based on the analysis of 42 cases of UECA from The Cancer Genome Atlas (TCGA) database, we conducted comprehensive profiling of the mutational landscape (supplementary material, Figure S2) [18]. Applying a mutation frequency cutoff of ≥10%, 31 high‐frequency mutated genes were identified. The most frequently mutated genes were PIK3CA (38.1%), KMT2C (21.4%), KRAS (19.0%), and TTN (19.0%). Missense mutations predominated, although frameshift, nonsense, splice site mutations, and multi‐hit events were also observed. Notably, the high‐frequency mutated genes are implicated in several critical signaling pathways, including PI3K/AKT/mTOR (PIK3CA), RAS/RAF/MEK (KRAS), HER2 signaling (ERBB2), and tumor suppressor genes (TP53, FBXW7, ARID1A).

Comparative evaluation of the gene mutation landscapes between 11 HPV‐associated CPCC cases and 42 HPV‐associated UECA cases from TCGA revealed that both subtypes exhibited significant molecular heterogeneity driven by recurrent somatic alterations; however, they displayed distinct profiles in mutation frequencies, key mutated genes, and implicated oncogenic pathways. Both HPV‐associated CPCC and UECA showed a high prevalence of genetic mutations, with at least one alteration identified in 81.8% (9/11) of CPCC and 97.6% (41/42) of HPV‐associated UECA samples; missense mutations were the predominant mutation type in both, accompanied by variants such as nonsense, frameshift, splice site, and multi‐hit events. Additionally, shared high‐frequency genes included KRAS (18.8% in CPCC versus 19.0% in UECA) and KMT2C (18.8% versus 21.4%), both implicated in critical signaling pathways, such as RAS/RAF transduction, which underscores a common role in driving cervical adenocarcinoma pathogenesis through dysregulated cellular proliferation and epigenetic modifications. However, key differences were evident: HPV‐associated CPCC exhibited unique high‐frequency mutations (≥18%) in AK1, ARHGAP39, KRT24, MICAL3, and SLC6A9 (each at 27.3%), with pathway enrichment highlighting AK1's involvement in adenosine metabolism and KMT2C in epigenetic regulation. In contrast, UECA displayed a broader set of recurrent mutations (≥10%), involving PIK3CA (38.1%), TTN (19.0%), ERBB2, TP53, FBXW7, and ARID1A, which are enriched in diverse pathways such as PI3K/AKT/mTOR (via PIK3CA), HER2 signaling, and tumor suppression, indicating a more extensive involvement of oncogenic networks. Furthermore, the mutation landscape in UECA demonstrates higher overall mutation rates and a greater diversity of altered genes, potentially reflecting differences in etiology or progression between subtypes.

### Proteomic profiling of DEPs in HPV‐associated CPCC

#### Integrated GO enrichment analysis of HPV‐associated CPCC pathogenesis

The volcano plot showed differential protein expression between HPV‐associated CPCC tissues and adjacent normal tissues, identifying 2,254 upregulated proteins (92.6%) and 181 downregulated proteins (7.4%) among all DEPs (supplementary material, Figure S3). GO enrichment analysis of DEPs from 11 CPCC cases revealed prominent functional changes in three categories. In biological process, enriched terms were mainly related to extracellular matrix remodeling, cell adhesion/migration, inflammatory/immune response, epithelial‐mesenchymal transition, and angiogenesis, suggesting enhanced invasiveness and tumor microenvironment remodeling. In cellular component, DEPs were mainly enriched in extracellular space, basement membrane, focal adhesion, and collagen‐containing extracellular matrix, indicating altered cell‐matrix interactions. In molecular function, the main enriched terms included heparin binding, integrin binding, growth factor activity, and receptor binding, supporting roles in signal interaction, cellular anchoring, and motility acquisition (supplementary material, Figure S4).

#### Proteomic identification of core DEPs and functional grouping analysis in HPV‐associated CPCC

**Figure 6:**
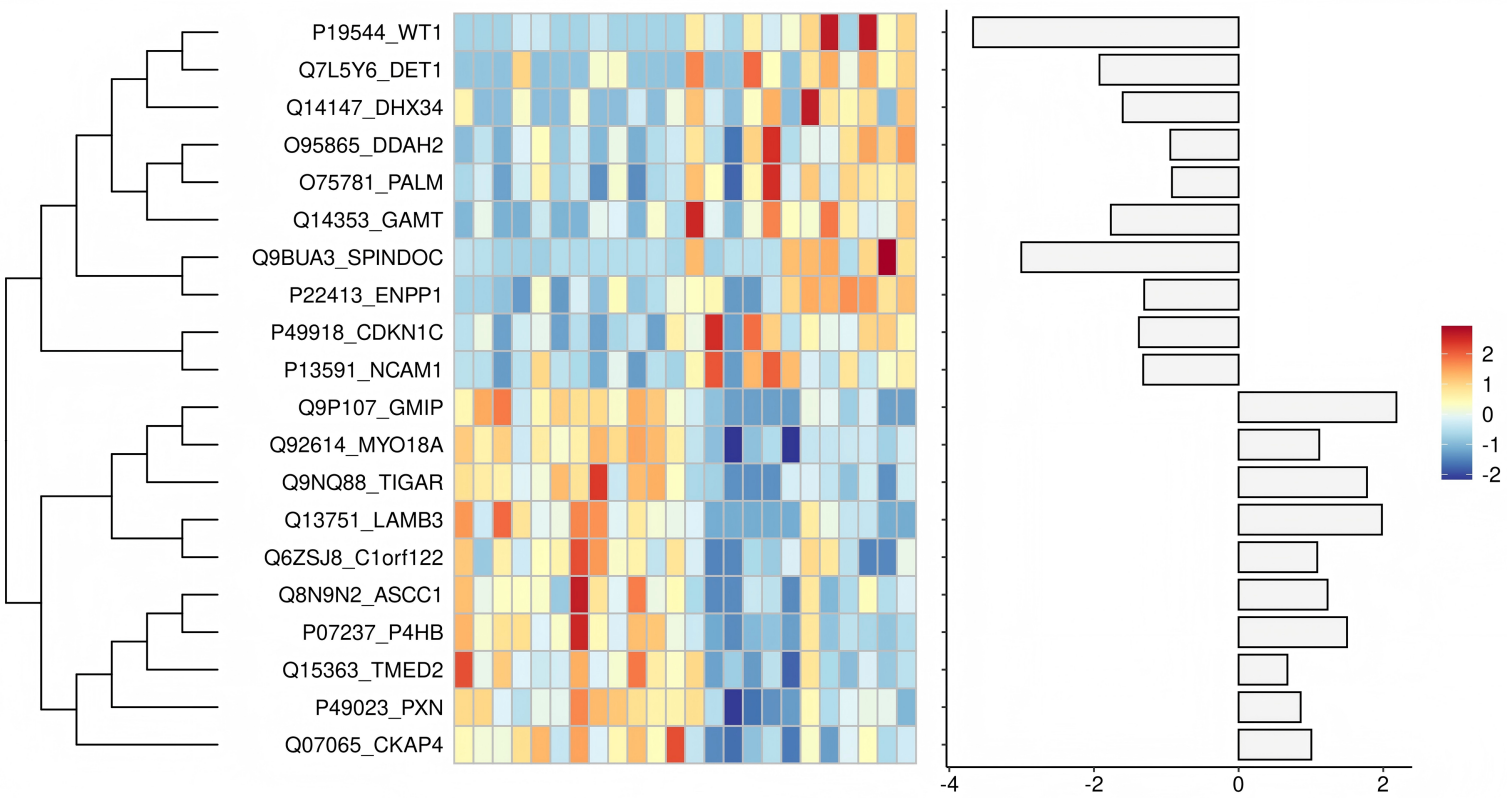
Proteomic identification of core differentially expressed proteins (DEPs).

Through proteomic analysis of paired tumor and adjacent nontumor tissues from 11 patients with CPCC, heatmap visualization successfully identified 20 core DEPs, comprising 10 significantly upregulated and 10 significantly downregulated proteins. Functional enrichment analysis of the 10 upregulated core proteins, categorized into two distinct groups: Group 1: GMIP, TIGAR, LAMB3, C1ORF122, and MYO18A (CD245); Group 2: ASCC1, P4HB, TMED2, PXN, and CKAP4, revealed that their aberrant expression was highly associated with CPCC malignant phenotype (Figure 6).

## Discussion

By integrating the clinicopathological, immunophenotypic, and molecular features of HPV‐associated CPCC, this study supports its characterization as an aggressive subtype of cervical adenocarcinoma. Analysis of our 16 cases together with 12 literature‐derived cases (total *n* = 28) further underscores its aggressive phenotype. The median age at diagnosis was 47 years, and vaginal bleeding was the predominant presenting symptom. Furthermore, HPV‐18 was the predominant genotype (86.7%, 13/15). This distribution aligns with the literature and suggests a potential tropism or enhanced oncogenic efficiency of HPV18 in driving this specific malignant phenotype. Diagnosis is typically made at advanced stages, with nearly two‐thirds of patients classified as FIGO stage III–IV, and the 3‐year OS rate is only 54.3%. Additionally, E‐cadherin expression was reduced or absent in 50% of cases, reflecting a loss of cell–cell adhesion that is closely associated with the tumor's invasive biological behavior.

The principal diagnostic challenge in confirming HPV‐associated CPCC lies in distinguishing it from metastatic gastrointestinal poorly cohesive carcinomas, due to their significant morphological overlap and variable IHC expression of gastrointestinal differentiation markers (e.g., CK20, CDX2, SATB2, and Villin). Key resolution strategies require: (1) comprehensive clinical evaluation using imaging (MRI/CT/ultrasonography) and endoscopic investigations to exclude metastasis of gastrointestinal poorly cohesive carcinomas; (2) p16/HPV status assessment, where diffuse strong p16 block positivity with confirmed high‐risk HPV infection strongly favors HPV‐associated CPCC, whereas gastrointestinal poorly cohesive carcinomas typically lack both features; and (3) interrogation of tissue‐specific markers such as Pax‐8, wherein positivity (albeit 20.0% in CPCC in the current cohort) argues against gastrointestinal metastasis. Furthermore, metastatic lobular breast carcinoma should also be considered in the differential diagnosis; however, these tumors typically express TRPS1, GATA3, ER, and progesterone receptor but lack both p16 overexpression and HPV infection. Another important differential diagnostic consideration is gastric‐type carcinoma with a poorly cohesive or even signet ring cell appearance; however, unlike CPCC, it is HPV‐independent and typically lacks block‐type p16 expression, which can aid in distinguishing the two entities. Finally, when diagnosing HPV‐associated CPCC, it is essential to differentiate it from CPCC‐like changes that arise in UCA after neoadjuvant therapy. Post‐neoadjuvant therapy tumors frequently exhibit treatment‐related necrosis and an acellular mucus lake, characterized by large tumor cells with abundant, intensely eosinophilic cytoplasm and visible pyknotic nuclei indicating apoptosis, typically accompanied by a significant inflammatory response.

The aggressive behavior and poor prognosis of HPV‐associated CPCC underscore the urgent need for biomarker‐driven therapeutic strategies. In this study, TF and Trop‐2 emerged as the most prevalent biomarkers, showing moderate‐to‐strong (2+/3+) expression in 92.3% and 85.7% of cases, respectively; nectin‐4 (42.9% with 2+ expression) and HER2 (33.3% with 1+/2+ expression) were also expressed in subsets of tumors. Given that ADCs targeting Trop‐2, nectin‐4, TF, and HER2 have already demonstrated clinical activity in cervical cancer and other solid tumors [19, 20, 21], the expression of these markers suggests potential eligibility for targeted therapies or ADCs, offering hope for CPCC treatment with the potential to improve survival outcomes. Additionally, PD‐L1 expression (CPS ≥1 in 31.3% of cases) identifies a subset of patients potentially responsive to immune checkpoint inhibitors.

WES revealed that HPV‐associated CPCC possesses a substantially distinct but partially overlapping genomic architecture compared with UECA, suggesting divergent pathogenic mechanisms. Notably, CPCC exhibits an exceptionally low tumor mutational burden (median: 1.28 mut/Mb), falling significantly below the 10 mut/Mb immunotherapy response threshold. HPV‐associated CPCC is also characterized by recurrent mutations in AK1, ARHGAP39, KRT24, MICAL3, and SLC6A9 (each 27.3%) – genes functionally linked to adenosine metabolism (AK1) and cytoskeletal dynamics (ARHGAP39, KRT24, MICAL3) [22, 23], with only partial overlap in KRAS (18.8% versus 19.0%) and KMT2C (18.8% versus 21.4%). In contrast, UECA prioritizes canonical oncogenic pathways through frequent PIK3CA mutations (38.1%; activating PI3K/AKT/mTOR), ERBB2 alterations (HER2 signaling), and tumor suppressor losses (TP53, FBXW7, and ARID1A). CNV further highlighted HPV‐associated CPCC‐specific biology, revealing recurrent MUC2 gains (63.6%), which potentially disrupt mucosal integrity, and bidirectional Y_RNA perturbations (54.5% gain/45.5% loss), suggesting noncoding RNA dysregulation.

Functional enrichment analysis of the 10 upregulated core proteins suggested two major themes: regulation of cell adhesion/migration and tumor metabolism/stress adaptation. Regarding adhesion and cytoskeletal dynamics, several upregulated proteins may contribute to the low‐adhesion phenotype and invasive behavior of the tumors. LAMB3, a key laminin component, is overexpressed and may impair basement membrane integrity while activating integrin signaling, thereby facilitating tumor cell detachment and local invasion [24]. PXN, a focal adhesion adaptor protein, enhances integrin‐mediated signaling and promotes actin cytoskeleton remodeling required for cell migration [25]. MYO18A may promote actomyosin contractility and directional migration in three‐dimensional matrices [26], and may further enhance migration through activation of RhoA/ROCK signaling via the Wnt/β‐catenin pathway [27]. GMIP, through negative regulation of Rho GTPases, may weaken intercellular adhesion and loosen cell junctions [28]. CKAP4, an ER‐microtubule anchoring protein, helps stabilize microtubules and may influence cell shape and motility [29]. In addition, several DEPs appear to be involved in metabolic reprogramming and adaptation to microenvironmental stress. TIGAR may promote tumor cell survival under hypoxic and nutrient‐deprived conditions through anti‐apoptotic effects [30]. P4HB, a key protein‐folding enzyme and marker of endoplasmic reticulum stress, reflects increased protein synthesis and sustained ER stress in tumor cells; by maintaining unfolded protein response homeostasis, it may support stress adaptation and contribute to extracellular matrix remodeling [31]. TMED2 participates in ER‐to‐Golgi vesicular transport [32], whereas ASCC1 is involved in transcriptional regulation and DNA damage repair, and together with C1ORF122 may contribute to genome stability surveillance [33, 34].

Despite defining the clinicopathological and molecular landscapes of HPV‐associated CPCC, this study has certain limitations. The relatively small cohort size, reflecting the novelty and rarity of this newly characterized entity, limits the statistical power for robust subgroup analyses and the generalizability of findings. A further limitation of this study is that, in a subset of advanced‐stage cases, morphologic evaluation was based on multiple‐site cervical biopsy specimens rather than radical resection specimens; although the biopsy material was considered adequate and representative for assessment, sampling bias and incomplete representation of the full tumor architecture cannot be entirely excluded. Additionally, although integrated genomic and proteomic analyses offered a broader understanding of the molecular features of HPV‐associated CPCC, the functional significance of the key alterations identified in this study was inferred primarily from *in silico* analyses and correlative evidence. Notwithstanding these limitations, HPV‐associated CPCC appears to represent an aggressive subtype of cervical cancer, characterized by a predominant association with HPV18, frequent presentation at advanced stage, poor survival outcomes, and genomic features distinct from those of UECA. In addition, ADC‐related markers, including Trop‐2, TF, HER2, and nectin‐4, may represent potential therapeutic targets in this disease.

## Author contributions statement

WL, X‐jW, Y‐mC, YS and DH performed study concept and design; WL, X‐jW, Y‐mC and DH performed development of methodology and writing, review and revision of the paper; JZ, WS, J‐cL, W‐xM, J‐cY, T‐mH and QX provided acquisition, analysis and interpretation of data, and statistical analysis; L‐bZ, XL and XW provided technical and material support. All authors read and approved the final paper.

## Supporting information


**Figure S1.** Copy number variation analysis on cancer‐related genes in 11 HPV‐associated CPCC
**Figure S2.** Mutational landscape of usual‐type endocervical adenocarcinoma in TCGA
**Figure S3.** Volcano plot analysis of differential protein expression in HPV‐associated CPCC
**Figure S4.** Integrated Gene Ontology (GO) enrichment analysis of HPV‐associated CPCC pathogenesis
**Table S1.** The clinicopathological features of HPV‐associated CPCC reported in the literature

## Data Availability

All data generated or analyzed during the current study are included in this published article.

## References

[1] Petersen Z , Jaca A , Ginindza TG , *et al*. Barriers to uptake of cervical cancer screening services in low‐and‐middle‐income countries: a systematic review. BMC Womens Health 2022; 22: 486. 36461001 10.1186/s12905-022-02043-y PMC9716693

[2] Castellsagué X , Díaz M , de Sanjosé S , *et al*. Worldwide human papillomavirus etiology of cervical adenocarcinoma and its cofactors: implications for screening and prevention. J Natl Cancer Inst 2006; 98: 303–315. 16507827 10.1093/jnci/djj067

[3] Haswani P , Arseneau J , Ferenczy A . Primary signet ring cell carcinoma of the uterine cervix: a clinicopathologic study of two cases with review of the literature. Int J Gynecol Cancer 1998; 8: 374–379.

[4] Nagtegaal ID , Odze RD , Klimstra D , *et al*. The 2019 WHO classification of tumours of the digestive system. Histopathology 2019; 76: 182–188. 31433515 10.1111/his.13975 PMC7003895

[5] Mariette C , Carneiro F , Grabsch HI , *et al*. Consensus on the pathological definition and classification of poorly cohesive gastric carcinoma. Gastric Cancer 2018; 22: 1–9. 30167905 10.1007/s10120-018-0868-0

[6] Bartley AN , Washington MK , Colasacco C , *et al*. HER2 testing and clinical decision making in gastroesophageal adenocarcinoma: guideline from the College of American Pathologists, American Society for Clinical Pathology, and the American Society of Clinical Oncology. J Clin Oncol 2017; 35: 446–464. 28129524 10.1200/JCO.2016.69.4836

[7] Chiba Y , Kojima Y , Yazaki S , *et al*. Trop‐2 expression and the tumor immune microenvironment in cervical cancer. Gynecol Oncol 2024; 187: 51–57. 38723340 10.1016/j.ygyno.2024.04.022

[8] Veras E , Srodon M , Neijstrom ES , *et al*. Metastatic HPV‐related cervical adenocarcinomas presenting with thromboembolic events (Trousseau syndrome): clinicopathologic characteristics of 2 cases. Int J Gynecol Pathol 2009; 28: 134–139. 19188822 10.1097/PGP.0b013e318186a83b

[9] Washimi K , Yokose T , Noguchi A , *et al*. Diagnosis of primary pure signet‐ring cell carcinoma of the cervix. Pathol Int 2015; 65: 393–395. 25736344 10.1111/pin.12275

[10] Sal V , Kahramanoglu I , Turan H , *et al*. Primary signet ring cell carcinoma of the cervix: a case report and review of the literature. Int J Surg Case Rep 2016; 21: 1–5. 26874582 10.1016/j.ijscr.2016.02.007 PMC4802128

[11] Cracchiolo B , Kuhn T , Heller D . Primary signet ring cell adenocarcinoma of the uterine cervix – a rare neoplasm that raises the question of metastasis to the cervix. Gynecol Oncol Rep 2016; 16: 9–10. 27331127 10.1016/j.gore.2016.01.004 PMC4899544

[12] Wang YC , Yu YL , Fan CW , *et al*. Primary signet ring cell carcinoma of the cervix: a case report with review of the literature. Taiwan J Obstet Gynecol 2019; 58: 46–50. 30638479 10.1016/j.tjog.2018.11.008

[13] Kawai S , Torii Y , Kukimoto I , *et al*. A case of primary signet‐ring cell carcinoma of the cervix containing full genome of human papillomavirus 16. Indian J Pathol Microbiol 2019; 62: 146–148. 30706882 10.4103/IJPM.IJPM_507_17

[14] Salmen N , LaBella D , Strumpf K , *et al*. A case of primary signet‐ring cell cervical carcinoma treated with chemoradiation, brachytherapy, and adjuvant hysterectomy. Case Rep Obstet Gynecol 2021; 2021: 5544015. 34987874 10.1155/2021/5544015 PMC8723868

[15] Purwoto G , Nuryanto KH , Wibowo TA , *et al*. Could combination of radical hysterectomy and radiation effective in the treatment of primary cervical signet ring cell carcinoma?: a rare case report. Int J Surg Case Rep 2022; 94: 107083. 35430518 10.1016/j.ijscr.2022.107083 PMC9038544

[16] Lazhar H , Slaoui A , Rostoum S , *et al*. Primary signet ring cell carcinoma of the cervix: about an uncommon case report. Int J Surg Case Rep 2023; 105: 107950. 36924600 10.1016/j.ijscr.2023.107950 PMC10025990

[17] Abad‐Licham M , Cotrina C , Guerrero A , *et al*. Primary signet ring cell carcinoma of the cervix: case report and literature review. Ecancermedicalscience 2024; 18: 1671. 38439801 10.3332/ecancer.2024.1671 PMC10911662

[18] The Cancer Genome Atlas Research Network . Integrated genomic and molecular characterization of cervical cancer. Nature 2017; 543: 378–384. 28112728 10.1038/nature21386 PMC5354998

[19] Vergote I , Colombo N , Falcón M , *et al*. Tisotumab vedotin as second‐ or third‐line therapy for recurrent cervical cancer. N Engl J Med 2024; 391: 44–55. 38959480 10.1056/NEJMoa2313811

[20] Powles T , Valderrama BP , Gupta S , *et al*. Enfortumab vedotin and pembrolizumab in untreated advanced urothelial cancer. N Engl J Med 2024; 390: 875–888. 38446675 10.1056/NEJMoa2312117

[21] Rugo HS , Bardia A , Marmé F , *et al*. Overall survival with sacituzumab govitecan in hormone receptor‐positive and human epidermal growth factor receptor 2‐negative metastatic breast cancer (TROPiCS‐02): a randomised, open‐label, multicentre, phase 3 trial. Lancet 2023; 402: 1423–1433. 37633306 10.1016/S0140-6736(23)01245-X

[22] Dzeja P , Terzic A . Adenylate kinase and AMP signaling networks: metabolic monitoring, signal communication and body energy sensing. Int J Mol Sci 2009; 10: 1729–1772. 19468337 10.3390/ijms10041729 PMC2680645

[23] Rajan S , Terman JR , Reisler E . MICAL‐mediated oxidation of actin and its effects on cytoskeletal and cellular dynamics. Front Cell Dev Biol 2023; 11: 1124202. 36875759 10.3389/fcell.2023.1124202 PMC9982024

[24] Tsuruta D , Kobayashi H , Imanishi H , *et al*. Laminin‐332‐integrin interaction: a target for cancer therapy? Curr Med Chem 2008; 15: 1968–1975. 18691052 10.2174/092986708785132834 PMC2992754

[25] Deakin NO , Turner CE . Paxillin comes of age. J Cell Sci 2008; 121: 2435–2444. 18650496 10.1242/jcs.018044 PMC2522309

[26] Buschman MD , Field SJ . MYO18A: an unusual myosin. Adv Biol Regul 2017; 67: 84–92. 28942352 10.1016/j.jbior.2017.09.005 PMC5807147

[27] Hsu RM , Hsie YJ , Yang TH , *et al*. Binding of the extreme carboxyl‐terminus of PAK‐interacting exchange factor β (βPIX) to myosin 18A (MYO18A) is required for epithelial cell migration. Biochim Biophys Acta 2014; 1843: 2513–2527. 25014165 10.1016/j.bbamcr.2014.06.023

[28] Aresta S , de Tand‐Heim MF , Béranger F , *et al*. A novel rho GTPase‐activating‐protein interacts with Gem, a member of the Ras superfamily of GTPases. Biochem J 2002; 367: 57–65. 12093360 10.1042/BJ20020829 PMC1222866

[29] Nikonov AV , Hauri HP , Lauring B , *et al*. Climp‐63‐mediated binding of microtubules to the ER affects the lateral mobility of translocon complexes. J Cell Sci 2007; 120: 2248–2258. 17567679 10.1242/jcs.008979

[30] Bensaad K , Tsuruta A , Selak MA , *et al*. TIGAR, a p53‐inducible regulator of glycolysis and apoptosis. Cell 2006; 126: 107–120. 16839880 10.1016/j.cell.2006.05.036

[31] Feng D , Wang J , Li D , *et al*. Targeting prolyl 4‐hydroxylase subunit Beta (P4HB) in cancer: new roads to travel. Aging Dis 2023; 15: 2369–2380. 38029391 10.14336/AD.2023.1126 PMC11567247

[32] Sun MS , Zhang J , Jiang LQ , *et al*. TMED2 potentiates cellular IFN responses to DNA viruses by reinforcing MITA dimerization and facilitating its trafficking. Cell Rep 2018; 25: 3086–3098.e3. 30540941 10.1016/j.celrep.2018.11.048

[33] Pan Y , Tan J , Wu C , *et al*. Pan‐cancer analysis reveals ASCC family promotes the cancer progression of lung adenocarcinoma. Sci Rep 2025; 15: 22799. 40594069 10.1038/s41598-025-03946-0 PMC12216297

[34] Edogbanya J , Tejada‐Martinez D , Jones NJ , *et al*. Evolution, structure and emerging roles of C1ORF112 in DNA replication, DNA damage responses, and cancer. Cell Mol Life Sci 2021; 78: 4365–4376. 33625522 10.1007/s00018-021-03789-8 PMC8164572

