# Supplementary material for: Clinicopathological and molecular characterization of HPV‐associated cervical poorly cohesive carcinoma: a rare aggressive entity
Source: J Pathol Clin Res. 2026 Jun 14;12(4):e70100. doi: 10.1002/2056-4538.70100 (PMC13265394; doi:10.1002/2056-4538.70100)
Supplement: Supplementary file 1 — Figure S1. Copy number variation analysis on cancer‐related genes in 11 HPV‐associated CPCC Figure S2. Mutational landscape of usual‐type endocervical adenocarcinoma in TCGA Figure S3. Volcano plot analysis of differential protein expression in HPV‐associated CPCC Figure S4. Integrated Gene Ontology (GO) enrichment analysis of HPV‐associated CPCC pathogenesis Table S1. The clinicopathological features of HPV‐associated CPCC reported in the literature [file CJP2-12-e70100-s001.docx]

**Clinicopathological and molecular characterization of HPV-associated cervical poorly cohesive carcinoma: a rare aggressive entity**

W Liu, X-j Wang, Y-m Cui *et al*. *J Pathol Clin Res* <https://doi.org/10.1002/2056-4538.70100>

**Supplementary Figures S1–S4**

**Supplementary Table S1**

*
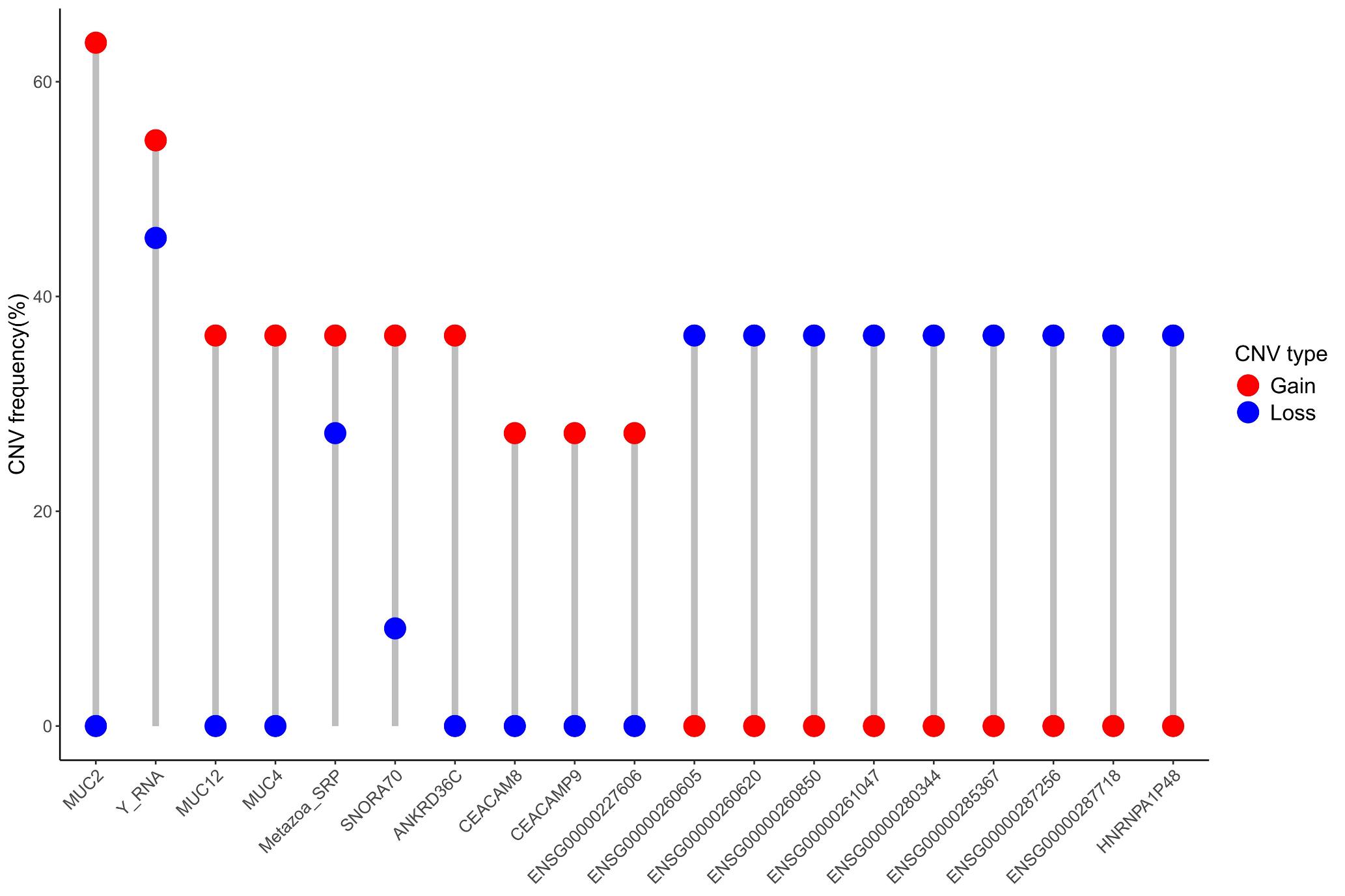
*

**Figure S1.** Copy number variation analysis on cancer-related genes in 11 HPV-associated cervical poorly cohesive carcinomas (CPCC).


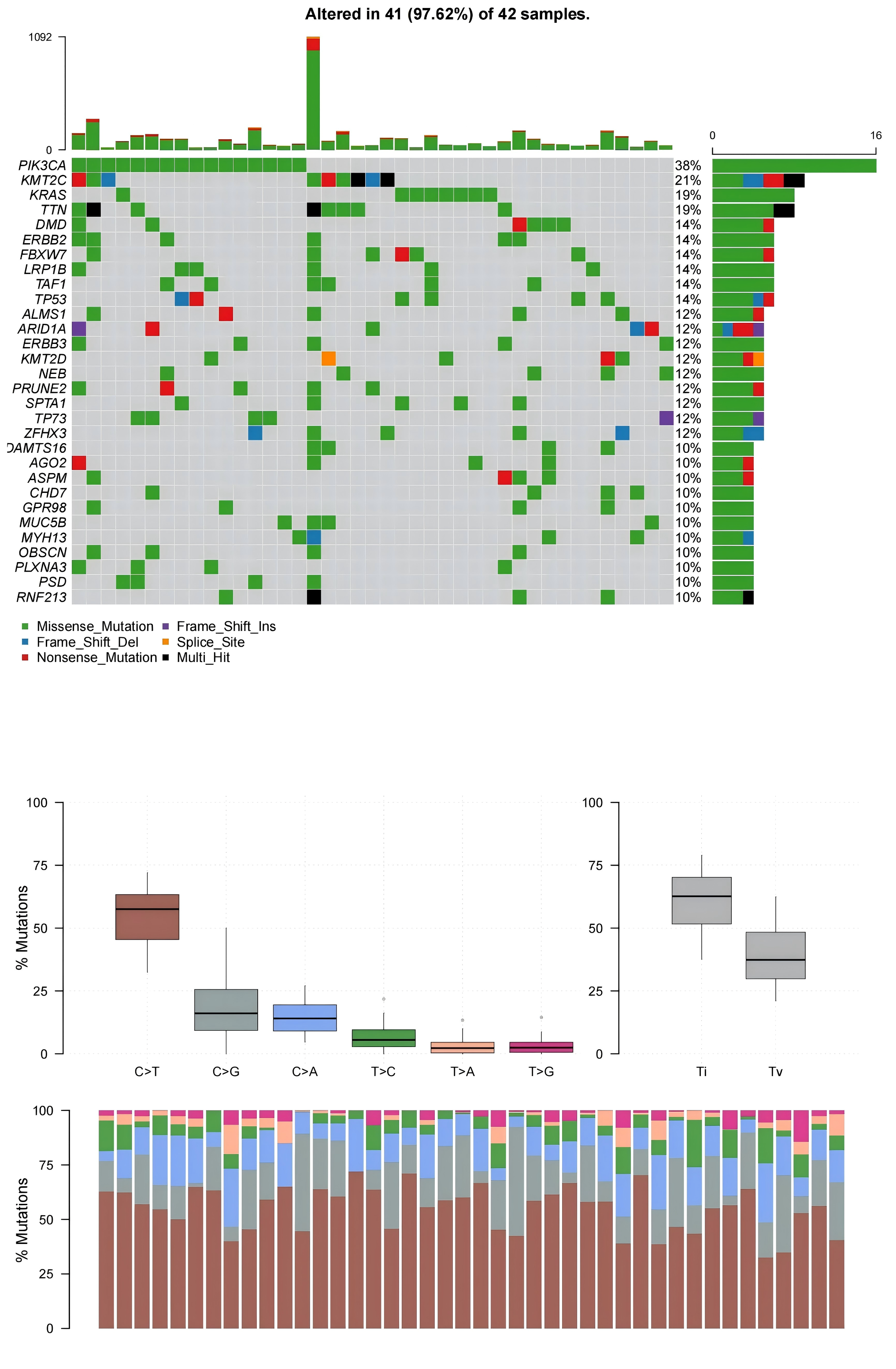
 **Figure S2.** Mutational landscape of usual-type endocervical adenocarcinoma in TCGA. **
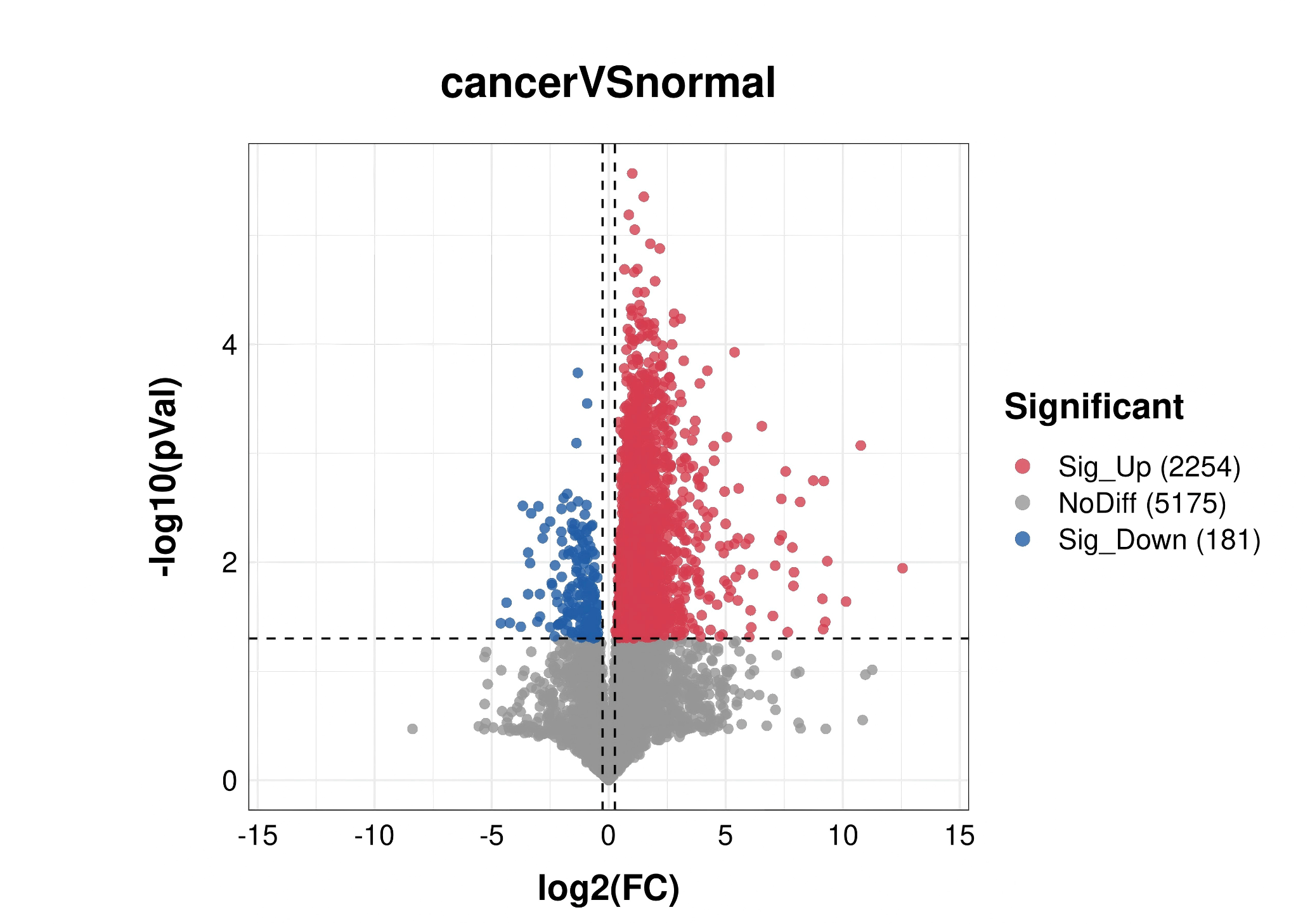
Figure S3.** Volcano plot a**nalysis of** differential protein expression in HPV-associated cervical poorly cohesive carcinomas (CPCC). The distribution of DEPs includes: 2,254 upregulated proteins (red dots), 181 downregulated proteins (blue dots).

**
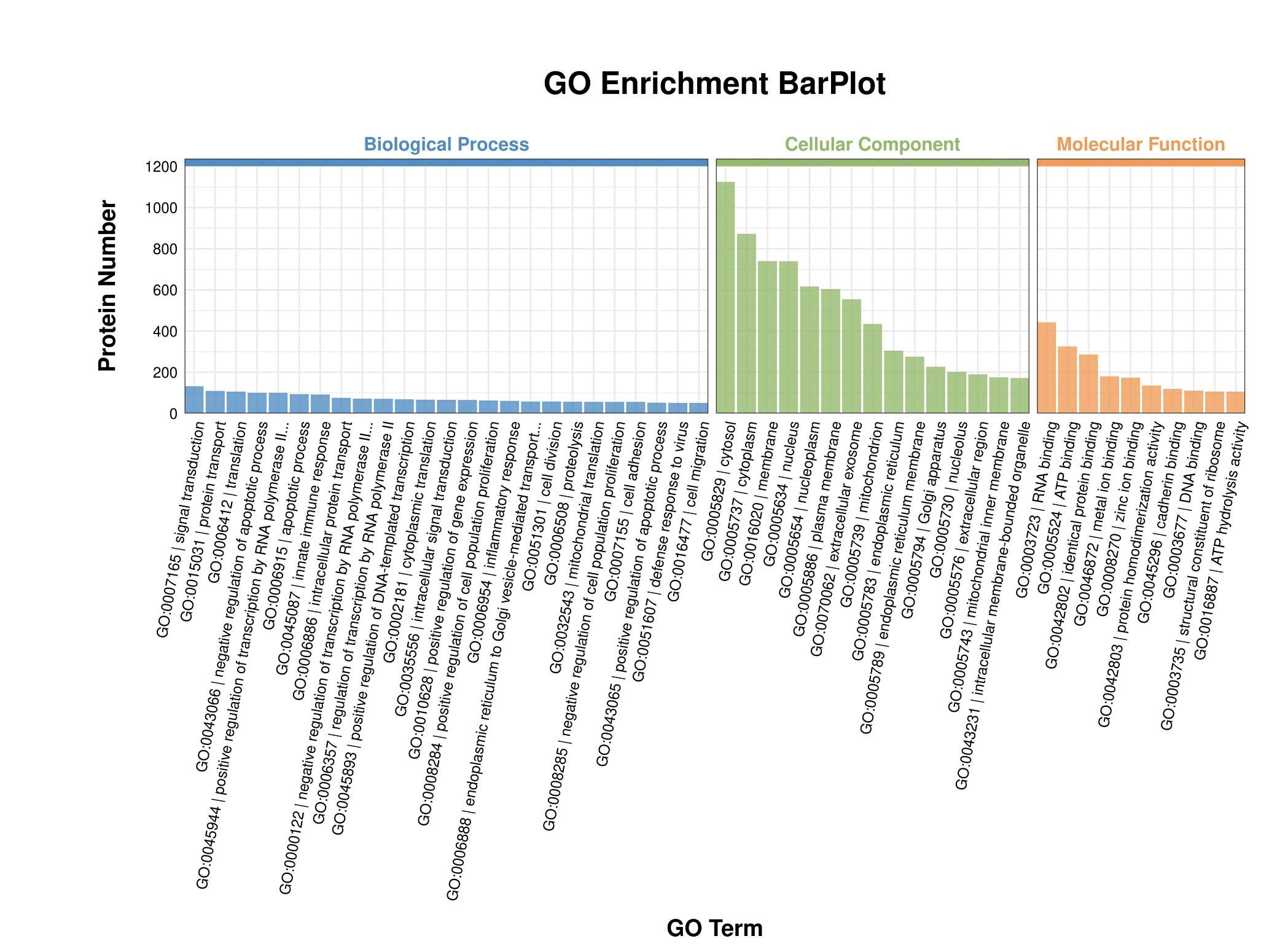
Figure S4.** **Integ**rated Gene Ontology (GO) enrichment analysis of HPV-associated cervical poorly cohesive carcinomas (CPCC) pathogenesis.

**Table S1.** The clinicopathological features of HPV-associated cervical poorly cohesive carcinomas (CPCC) reported in the literature

| **Case number** | **Reference** | **Age** | **Presentation** | **HPV/P16** | **Tumor size (cm)** | **Distant metastasis (M)** | **Other organ involvement** | **FIGO 2018 stage** | **Treatment** | **Follow-up** |
| --- | --- | --- | --- | --- | --- | --- | --- | --- | --- | --- |
|  |  |  |  |  |  |  |  |  |  |  |
| 1 | [3] | 38 | Postcoital vaginal bleeding | HPV18 | 3.5 | M0 | None | IIIC1 | Surgery followed by RT | 9 mo, ANED |
| 2 | [8] | 36 | Thromboembolic events | HPV+ | 2 | M1 | Cervical lymph nodes, lung | IVB | CT | 7 weeks, DOD |
| 3 | [8] | 43 | Left neck pain and swelling, Thromboembolic events | HPV18 | NR | M1 | Ovary | IVB | CT | 2 mo, DOD |
| 4 | [9] | 31 | Vaginal bleeding | HPV18 | 1.5 | M0 | None | IIA | Surgery followed by CT | 41 mo, ANED |
| 5 | [10] | 48 | Vaginal bleeding | HPV18 | 2.5 | M0 | None | IB2 | Surgery | 18 mo, ANED |
| 6 | [11] | 64 | Abdominal fullness | P16+ | NR | M1 | Cervical Lymph Nodes | IVB | Palliative treatment | 3 mo, DOD |
| 7 | [12] | 48 | Vaginal bleeding | P16+ | NR | M1 | Bilateral lower lung; appendix | IVB | Surgery followed by CT | 8 mo, AWD |
| 8 | [13] | 40 | NR | HPV16 | 3.8 | M0 | None | IB1 | Surgery + CT | 33 mo, ANED |
| 9 | [14] | 50 | Vaginal bleeding | P16+ | 7 | M0 | None | IB3 | Surgery followed by CT, RT | 12 mo, ANED |
| 10 | [15] | 39 | Postcoital vaginal bleeding | P16+ | 3 | M0 | None | IB2 | Surgery followed by CT, RT | 12 mo, ANED |
| 11 | [16] | 68 | Pelvic pain evolving for 4 months | P16+ | 5.4 | M0 | Bladder, urethra | ⅣA | CT | 1 mo, DOD |
| 12 | [17] | 51 | Vaginal bleeding | P16+ | 7 | M0 | None | ⅢB | without treatment | 1 mo, DOD |

+, positive; ANED, alive with no evidence of disease; AWD, alive with disease; CT, chemotherapy; DOD, dead of disease; LTF, lost to follow up; mo, month/months; NA, not available; NR, not reported; RT, radiotherapy.
